# Psychosocial consequences of potential overdiagnosis in prostate cancer a qualitative interview study

**DOI:** 10.1080/02813432.2020.1843826

**Published:** 2020-11-26

**Authors:** Sigrid Brisson Nielsen, Olivia Spalletta, Mads Aage Toft Kristensen, John Brodersen

**Affiliations:** a Department of Public Health, University of Copenhagen Faculty of Health Sciences, Section of General Practice & Research Unit for General Practice, Copenhagen, Denmark; b Primary Healthcare Research Unit Region Zealand, Sorø, Denmark; c Brandeis University, Waltham, MA, USA; d Southern Køge Medical Centre. Region Zealand, Køge, Denmark

**Keywords:** Qualitative research, prostate-specific antigen, prostatic neoplasms, medical overuse, quality of life

## Abstract

**Background:**

Prostate cancer is a frequently diagnosed cancer and made up 6% of male cancer deaths globally in 2008. Its incidence varies more than 25-fold worldwide, which is primarily attributed to the implementation of the prostate-specific antigen (PSA) test in developed countries. To reduce harm of overdiagnosis, most international guidelines recommend surveillance programmes. However, this approach can entail negative psychosocial consequences from being under surveillance for an (over)diagnosed prostate cancer.

**Aim:**

To explore men’s feelings and experiences in a surveillance programme.

**Design and setting:**

Qualitative study with Danish men diagnosed with asymptomatic prostate cancer Gleason score ≤ 6, who are in a surveillance programme

**Methods:**

12 semi-structured, individual interviews were conducted and analysed with systematic text condensation and selected theories.

**Results:**

Most informants reported that they were astonished at the time of diagnosis. They were aware of the small likelihood of dying from cancer, but in some cases, the uncertainty created ambivalence between knowing and not knowing. The men expressed their risk awareness in different ways: a realization that life does not last forever, uncertainty towards the future, a feeling of powerlessness, and a need for control.

**Conclusions:**

The men in this study had substantial psychosocial consequences from being labelled with a cancer diagnosis. Bearing these men’s high risk of overdiagnosis in mind, it is important to discuss whether the harms of this diagnosis outweigh the benefits. The psychosocial consequences of being in a prostate cancer surveillance programme should be explored further.

**KEY POINTS**
- Current awareness: The number of men living with an asymptomatic prostate cancer has increased the last 20 years after the implementation of the PSA test.
- Main Statements:
  Men living with an asymptomatic, low-risk prostate cancer experience negative psychocosial consequences
- GPs should consider the possible negative psychosocial consequences in their decision-making of measuring the PSA level

## Introduction

Prostate cancer is the second most frequently diagnosed cancer accounting for 14 % of total new cancer cases globally [1]. Incidence of prostate cancer varies more than 25-fold worldwide with highest rates in Europe, Oceania and North America [1].

This variation is primarily caused by the implementation of the prostate-specific antigen (PSA) test in the early 1990s. The PSA test can detect clinically important tumours but also finds indolent cancers that otherwise might not have been detected [1]. A Cochrane review of five randomized control trials found that screening with PSA tests had no significant impact on prostate-specific mortality [2]. Most clinical guidelines are hesistant in recommending PSA-testing but in general practice PSA testing is recommended if a male patient present with lower urinary tract symptoms (LUTS). However, there is no causal relationship between LUTS and clinical significant prostate cancer [3]. Therefore, PSA screening and such non-evidence based clinical guideline recommendations can lead to substantial overdiagnosis of prostate cancer.

In the context of prostate cancer, overdiagnosis – or overdetection – is the identification of indolent neoplastic pathology that was never going to cause harm, pathology that does not progress, that progress too slowly to cause symptoms or harm during a person’s remaining lifetime [4]. In patients with a localised prostate cancer of a Gleason score 6 the probability of cancer metastasizing is small, as approximately 90 % is assumed to be overdiagnosed [5].

Overdiagnosis of prostate cancer can cause harm in many ways. Common major physical harms include overtreatment, infection, blood loss requiring transfusion, erectile dysfunction and incontinence [2,6]. The purpose of surveillance programmes is to diminish or prevent harms of overdiagnosis and overtreatment. Therefore, most international guidelines recommend Active Surveillance (AS) or Watchful Waiting (WW) depending on Gleason score, age and anticipated life expectancy [7,8].

There are few qualitative studies about psychosocial aspects of being diagnosed with prostate cancer: a Swedish study with stricter inclusion criteria than the present study [9] and one from the USA that included a psychosocial intervention of diet, exercise, stress management practices and group support [10]. Several quantitative studies have measured psychosocial or quality of life aspects of being diagnosed with prostate cancer. However, most of these studies used questionnaires with low content validity [11]. None reported questionnaires’ statistical measurement properties (psychometrics) [12–18]. After scrutinising previous studies we found missing knowledge about potential unintended psychosocial harm.

### Aim

This study explores the feelings and experiences of men diagnosed with a localised prostate cancer Gleason score ≤ 6 in a psychosocial context.

## Design, material and methods

Informants were recruited from Department of Urology, Zealand University Hospital either by receiving an invitation letter from a nurse when visiting the out-of-hours clinic or by mail.

SBN accessed the medical files and identified 10 patients in AS, 10 patients in WW and 17 newly diagnosed patients in AS or WW. Informants in WW had never received treatment for their prostate cancer. Informants in both AS and WW had regular outpatient clinic visits every 6–12 months including PSA-test and meeting an urologist. Patients were included if they had a Gleason score ≤ 6 and no serious co-morbidities such as cardiovascular disease, other cancer, psychiatric disease etc. 37 invitation letters were sent in the first half of 2017. Three weeks later, non-responders received a reminder (see Table 1). The invited men were asked to contact SBN or JB if they wished to participate. During the interview period, 15 men accepted to participate in an interview; however after 12 interviews we assessed that data saturation was achieved and no further interviews were conducted.

**Table 1.** The informants are called N together with a number representing the chronological order of the interviews.

Informant	Age (year)	AS/WW	Time with PC	Work status	Previously profession	Marital status	Residence	Interviewer	Place for interview
N1	68	AS	3 years	Retired	Phd. Degree	Married	Town	JB/SBN	Home
N2	73	AS	3 years	Retired	Policeman	Married	City	JB/SBN	JB's office
N3	74	AS	7 years	Retired	Self-employed	Relationship	City	JB/SBN	Home
N4	70	AS	3 years	Retired	Salesman	Divorced	Town	SBN	Home
N5	77	WW	10 years	Retired	Unskilled work	Divorced	City	SBN	Home
N6	81	WW	12 years	Retired	Merconom	Divorced	City	JB/SBN	Home
N7	76	WW	2 years	Retired	Leader-post in the Navy	Married	Village	JB/SBN	Home
N8	79	WW	4 years	Retired	Leader-post in a bank	Married	Town	SBN	Home
N9	78	AS	5 months	Retired	Farmer	Married	City	JB/SBN	JB's office
N10	72	AS	3 months	Retired	Auto mechanic	Married	City	JB/SBN	JB's office
N11	74	AS	7 months	Retired	Teacher	Married	City	SBN	Home
N12	73	WW	7 months	Retired	Teacher	Married	Town	SBN	Home

‘AS/WW’ is Active surveillance (AS) or Watchful waiting (WW). ‘Time with PC’ is the number of years since the informant got the prostate cancer diagnosis. Interviews were conducted either by both J. Brodersen (JB) and S.B. Nielsen (SBN) or by SBN alone..

Twelve semi-structured, individual interviews were conducted from February-June 2017 and lasted between 50 and 120 min. The interviews were audiotaped and transcribed by SBN. The interview guide was developed by examining relevant themes from previous studies on this topic [10,19,20]. The interview guide was continuously adjusted by SBN and JB by thematic condensation between the interviews (see Appendix). The conceptualisation of ‘psychosocial consequences’ was based on the biopsychosocial model in which people are not regarded as passive victims but as conscious individuals who are aware of their surroundings and react to them [21].

During this study, JB adapted the Consequences of Screening questionnaire for use in men diagnosed with prostate cancer [22]. A preliminary draft version of the questionnaire was handed out when the interviewer had assessed that the semi-structured interview was completed. In some cases, completing the questionnaire inspired the men to continue the interview. This questionnaire has not been published yet, but can be required upon request.

In Denmark, qualitative studies in which informants are anonymized are not considered for approval by The Danish Data Protection Agency or the local committee of research ethics.

After several readings of the transcripts, the informants’ thoughts, feelings and experiences were gathered in thematic units of meaning through systematic text condensation analysis. Quotes were used as examples of these thematic units and were selected by SBN, OS and JB. Themes in the systematic text analysis was compared to existing litterature in the field; Festingers theory regarding Cognitive Dissonance, Sontag’s ‘Illness as Metaphor’ and the PhD dissertation ‘Aiming for the Ordinary’ by Offersen. This comparison and selection of theory was done by SBN, OS and JB. The research team consisted of a medical student (SBN), a PhD student in anthropology (OS), and two GP’s of which two have a PhD degree and one is a professor in general practice (MATK)(JB). SBN and OS have Danish and English respectively as native languages and translated quotes.

## Results

The majority of the twelve informants said they were surprised when they received the diagnosis. In most cases they had asked their GP for the test themselves but not because they had experienced any symptoms. On one hand, informants were aware of the low risk of mortality combined with this type of cancer. On the other hand, they also knew that there were no guarantees. Their awareness of risk was expressed in different ways: e.g. the realization that life does not last forever, uncertainty towards the future, a feeling of powerlessness and a need for control. Some men expressed an ambivalence towards knowing or not knowing about the cancer. These findings will be elaborated below. In the interviews there were no sign of thematic difference between informants in AS and informants in WW.

### Text condensation analysis

#### Reactions to the diagnosis

The informants generally had two types of reactions to getting the diagnosis: either they were shocked or they reacted with a ‘that’s just the way it is’ attitude. Many of the men were also shocked because they did not feel sick. They were not experiencing any symptoms but suddenly were told they had a cancerous disease.

…when she says, well starts by saying, you have cancer, right. Well then I thought.. It was just like the carpet was pulled out from under me. Because I wasn’t prepared for it at all and I really hadn’t expected it, because I feel strong and healthy. And I’m active and feel better than many people my age and my friends … so it was a big surprise to me and it also meant that I had a hard time getting over it. I couldn’t sleep at night because thoughts were running around in my head. And I thought what now? What should I do.. should we move away from here? I was thinking about all different kinds of scenarios, yeah? N11

#### Life does not last forever

Getting the prostate cancer diagnosis confronted informants with the fact that life does not last forever. In some cases it made them prepare in a practical way, e.g. cleaning out the storage room, travelling to a place they always wanted to see or just wanting to make the best out of the time that was left. An enhanced appreciation of life was also one of the ways this realization was expressed.

It’s not that I think about it every single day but.. it’s involuntary… The circumstances, as I see it, there are more of them. The more circumstances that can be the end of your life the greater chances are there that it will end. Before you just thought.. Well you didn’t really think about it at all. But then it got its foot in the door and if some disease comes, it might go fast. N8

#### Uncertainty towards the future

Most men initially described themselves as being calm and cool when dealing with their prostate cancer. They knew from conversations with their doctors that they had a low-risk cancer but the fact that no one was able to guarantee that *their* cancer would not develop made them feel uncertain about what the future might bring. Some described this uncertainty as being in the back of their minds, surfacing when they were reminded of the cancer in some way. This could be through TV-shows or when time came for their regular PSA test.

It’s not something that has affected me, it’s not something I feel. So… I’m at ease about it. But it can quickly change, one should be clear on that, right? N3

#### Relations

Many of the men talked to their partners or friends about their disease. There were many examples of men who talked to friends in similar situations about their prostate cancer. Many felt that having a prostate cancer diagnosis had given them an enhanced understanding of others who were sick as well. Men who told us they were not emotionally affected by the prostate cancer diagnosis could easily imagine how it might affect others in their position.

Yes sure, it’s something that stays with you and that you have to think about and deal with it. It’s a matter of coming to terms with reality, but it’s a big change in reality so some people get depressed about it. I understand that. N1

#### A new meaning to bodily sensations

Some men experienced normal bodily sensations as a reminder of their prostate cancer. One man thought of his prostate cancer every time he had to pee due to his LUTS. Another man occasionally felt pain around his rectum (which he attributed to hemorrhoids), but which nonetheless made him fear his cancer was spreading. One asked his doctor where the cancer would spread first if it did spread. The doctor answered the lymph nodes and afterwards the informant started checking his lymph nodes on a regular basis.

Then you notice even more, have your ear to the ground, exactly, if you feel something in the lower abdomen, right? Is this that? Is this that? Because of (the diagnosis). N8

#### Powerlessness

Many of the men asked their doctors how to improve their odds. The fact that there was nothing they could do gave them a feeling of powerlessness. Nonetheless some men changed their behaviour to try to improve their odds. One man was a handball referee in his spare time and continued with this to be physically ready if he should suddenly need of surgery for his prostate cancer. Another man ate buckwheat porridge every morning because he was told it was good for fighting cancer.

Yeah, there’s not much I can do about this, right. Even if I want to I don’t. Although I think there might be a power of the will in those cases; I think that helps people. I’m not saying get cured but at least put up with it. N1

#### Regular testing

Due to serious side effects of treatment for prostate cancer, the men who get this type of prostate cancer are advised to participate in surveillance programmes rather than treatment. Surveillance programmes consist of regular PSA blood tests, digital rectal exams and depending on type of surveillance programme some biopsies. Going to regular checks and staying in contact with the health system gave the men a feeling of safety and control, but waiting for PSA results were also a cause for anxiety and a reminder of the prostate cancer. For some men, the PSA value was more or less constant. For others, the PSA rose and fell without any specific explanation or consequence. In spite of this none of the men considered not going. Follow-ups were the mens’ safety net. Not going was not an option.

It’s not that I don’t want to go there, I know I have to go, and I’m going. But it does create some anxiety because of the uncertainty, yeah there is some of that definitely. I’m sure it can’t be avoided actually. N1

#### It’s good to know – or is it?

In spite of concerns that came with their diagnosis the informants thought it was good to know. They could not be certain the cancer would not disseminate and therefore keeping an eye on it was important. They also thought of cancer as something you have to catch in time. If they did not know about the cancer they could risk that when their cancer was finally discovered, it would be too late. These men knew that the odds of this happening were very slim but they were not willing to risk it for a life of happy ignorance.

No, certainly not. Because.. Well.. It is true that I would have lived in ignorance and I would have lived happy with that. But if it had spread, without my knowing. That’s why I say, oh well I don’t mind having those biopsies, they’re not that bad. N8

### Theoretical analysis

Leon Festingers’ Cognitive Dissonance Theory [23] describes how experiencing discrepancy between what you know and what you do can cause one to feel a sense of disharmony (dissonance). People seek harmony by having continuity between what they know, feel and do (consonance). Festinger writes that the same thing can happen when you make a decision. If two options are both good, it will create dissonance. Studies show that when people are forced to choose, different mechanisms are in play to reduce dissonance: the first step can be to rewind your decision. If that is not possible, the next step would be to create new cognitions that support the option you choose as superior. Last, you can make the options look alike so that it does not matter which one you choose. In most cases, getting the PSA test was the informant’s own choice. They went to the doctor and asked for it. In this situation, the decision cannot be undone– Pandora’s Box had been opened.

An example of a cognition we meet was that the risk of the disease was exaggerated. The men in this study had a very slow growing prostate cancer that would probably never develop. So describing it as a ‘bomb’ that suddenly makes you ‘deathly ill’ would probably be exaggerating its potential.

Well it… It can seem like a bomb, suddenly one day you are critically ill. Maybe it’s past where it can be fixed. And has spread. N7

When the risk of disease is exaggerated by informants, the hazards of knowing versus not knowing look alike. Likewise, if you feel fine and believe the diagnosis does not affect you, then it does not matter that you have to live with the knowledge because you made the decision to know about it.

Yes, well you might say I’m already in the system. Of course it would be nice if you were free from it, but I’m in the system and well, now there’s somebody keeping an eye on it and I’m fine with that. N4

The PhD dissertation ‘Aiming for the Ordinary’ by Offersen looked into bodily sensations in everyday life in the Danish middle class: ‘We believe that it is in the uncertain and ambiguous ‘What if?’ of sensation experience that socio-cultural context, subjectivities and the pervading discourse of biomedicine interweave and produce meaning to bodily sensations and configure symptoms’ [24]. Offersen wrote about a woman named Ingrid, who had a brown spot removed. It turned out there was cancer in the brown spot and afterwards Ingrid was more observant towards her other spots. The ‘What if?’ was now closer to ‘a symptom of illness’. Some of the men we talked to had the same experiences: the ‘What if?’ in the bodily sensation made them think of their cancer. Therefore, a sensation that usually would have been dismissed as ordinary suddenly took on a new meaning, as the men wondered, ‘What if the prostate cancer has spread’.

In ‘Illness as Metaphor’, Sontag wrote that the controlling metaphors in descriptions of cancer were drawn from warfare. Cancer cells do not simply multiply, they are *invasive*. Treatment aims to *kill* cancer and there is a *war on cancer* in our society. [25]. Therefore, through the metaphors of cancer we are not just giving these men a diagnosis of prostate cancer. We are sending them into war. However, the surveillance programmes are not like a regular cancer treatment where we can *fight* and *kill* the cancer cells. In the case of these particular men, it might be more accurate to say that they find themselves in a cold war where they never know when the enemy will strike, but they have to be ready if or when it happens.

Despite their vigilance, the men we talked to also felt powerless because they believed there was nothing they could do. They could not make the enemy go away, and going into war (treatment) would have too many costs (side effects). Therefore, like rationalizing and building weapons, the men prepared their lives and bodies, e.g. writing wills, cleaning out storage rooms, staying active. When hearing noises, their first thought was ‘is the enemy here now?’ or in other words: Is this sensation the prostate cancer spreading?

## Discussion

### Summary

Being diagnosed with prostate cancer affected the mens’ lives in different ways. Many informants told us they were surprised to get a prostate cancer diagnosis because they felt healthy. In most cases they asked for the PSA test themselves but had been asymptomatic. The informants knew that their type of cancer was indolent and that they would probably die with it, not from it. Yet there were no guarantees, which in some cases was cause for ambivalence towards knowing versus not knowing. Getting the prostate cancer diagnosis made some aware that life does not last forever. It was also cause for uncertainty about what the future might hold and created a feeling of powerlessness and a need for control.

### Methodological considerations

In order to get a comprehensive understanding of our research question, we decided to use a semi-structured interview approach given that it is well-suited when the subject is sensitive as we expected this to be. Informants were encouraged to talk freely, and experiences, thoughts and feelings were pursued with elaborating questions. The setting was chosen by the informants in order to cause the least discomfort, which typically meant that the interviewer(s) travelled to the participant’s home. All participants were informed that they would be anonymized by letter.

We chose to exclude men with other serious conditions because we did not want psychosocial consequences from other conditions or severe co-morbidity to influence our results. When in doubt about whether to include an informant due to a condition, we used the pre-interview telephone call to explore the seriousness of this disease.

As previously mentioned, in some interviews the introduction of a questionnaire had an inspirational effect on the men. During the study, we started to hand out the questionnaires earlier in the process if the conversation was not flowing. The themes in the interview guide and questionnaire were comparable and therefore the content of the conversation was the same even though the interview technique changed.

In some of the interviews, the subject of whether or not it was the right decision to have had the PSA test came up. Questions may arise about whether it is ethical to have this conversation with the informants, as they cannot go back in time to choose differently, and in most cases they requested the test themselves. As interviewers we were very cautious not to bring this issue up in the conversation unless the informant brought it up himself. In that way we believe that we did not plant thoughts or feelings in the patient that were not already there. Nonetheless we are also aware that it is possible to influence the informants in some way just by engaging in their stories.

### Comparison with existing literature

In our study, we found that informants generally reacted to the diagnosis in one of two ways: they either reported feeling shocked or they reacted with a ‘that’s just the way it is’ attitude. Similar findings have been made in a Danish PhD thesis about mammography screening [26] and in the qualitative intervention study by Kronenwetter [10], where a reaction of shock and worry or ‘bump in the road’ attitude was described.

In our study, informants reported a feeling of powerlessness, which has also been described in a study by Brodersen et al. investigating consequences of screening for lung cancer and in a study by Hansson et al. about men diagnosed with asymptomatic aortic aneurysm [19,22].

All informants expressed that the diagnosis forced them to confront the notion that ‘life does not last forever’. This was shown in informants’ renewed appreciation for life and thoughts about death. This was also seen in Kronenwetter [10] and Hansson et al. [19].

PSA-testing was both connected with anxiety and a feeling of control. Informants generally had faith in the health system and trusted their physician to keep their prostate cancer in check. This experience was also found in men from the Hedestig study [9].

In the Hedestig study [9], having untreated localised prostate cancer was equated with having problems being sexually active. In our study, only one informant reported a difference in his sexual life after the diagnosis. This is similar to Hedestig’s [9] findings where the men described their manhood as ‘being restricted’. In Hedestig [9] the men expressed that they preferred discussing their impotence with other men. In our study, a woman (SBN) was present in all interviews and this could have had an influence on our findings in this particular area.

The finding of participants’ ambivalence and dilemma between knowing and not knowing, has also been seen in the study by Hansson [19].

In our study, we found that the lack of knowledge about what the future might bring created a great deal of uncertainty. Hansson also found that many informants experienced anxiety and worry due to uncertainty regarding their diagnosis. Informants who felt healthy previous to the diagnosis now felt fragile and old [19].

### Limitations

We have no information on the people who declined our invitation besides that they matched our inclusion and exclusion criteria.

One of the informants had been cured of another cancer diagnosis 5 years before, but it was not noticed before the interview. We only included interview parts where this man specifically talked about prostate cancer in the analysis.

It could be argued that the men we talked to are a select group. It is well known that people who participate in research projects typically belong to a more robust part of the general population. This combined with the fact that these men are elderly without other serious conditions could add to this tendency.

### Implications for research

The men participating in this study reported substantial negative psychosocial consequences after being diagnosed with a localised prostate cancer Gleason score ≤ 6. Future research is needed to investigate the degree and duration of these consequences.

### Implication for practice

GPs should be aware of the unintended harm that can result from an overdiagnosed cancer and the risk of overdiagnosis when using the PSA test in clinical settings. This awareness can make the GP better equipped to give nuanced advice about the PSA test in the future.

## Interview Guide

Used in the qualitative study:

‘Psychosocial Consequences of Potential Overdiagnosis in Prostate Cancer’

- Feelings about discovering the cancer
- Feelings about living with a cancer diagnosis
- Changed self-perception of one’s health
- Communication with health professionals (good/bad?)
- A feeling of having something that needs to be fought – fighting gives a feeling of control
- A constant threat
- Uncertainty/no control of one’s situation
- A fear of the unknown
- Loneliness
- Fear
- Worry
- Anger
- Frustration
- Irritability
- Anxiety
- Mood swings
- Lack of confidence
- Sad/depressed
- Hard to express one’s feelings
- Thoughts about/fear of death
- Spirituality
- Fragility of life
- An enhanced appreciatione of life
- Life does not have to be as long as possible (quality vs. quantity)
- Pressure from surroundings to undergo treatment
- Pressure from surroundings to talk about your current situation
- Lack of support/communication
- Surroundings being worried/overprotecting
- Feeling alone with the disease/social distancing
- Comparing to others in a similar situations
- Partner/spouse is also affected by the situation/the diagnosis
- Challenges in the relationship
- Less sexually active than before diagnosis, lower masculine self esteem
- Decreased sexual desire
